# Unusual Endoscopic Findings in Children: Esophageal and Gastric Polyps: Three Cases Report

**DOI:** 10.1097/MD.0000000000002539

**Published:** 2016-01-22

**Authors:** Smaranda Diaconescu, Ingrith Miron, Nicoleta Gimiga, Claudia Olaru, Ileana Ioniuc, Iulia Ciongradi, Ioan Sarbu, Gabriela Stefanescu

**Affiliations:** From the Grigore T. Popa University of Medicine and Pharmacy (SD, IM, NG, CO, II, IC, IS, GS); St. Mary's Emergency Hospital for Children (SD, IM, NG, CO, II, IC, IS) and St. Spiridon Emergency Hospital, Iasi, Romania (GS).

## Abstract

Isolated polyps of the upper digestive tract are rarely diagnosed in children, being usually an incidental finding during endoscopic exploration.

The diagnostic, therapy, and outcome of these lesions are based on endoscopy and pathology.

In a 5-year period, clinical features, topography, size, pathology, therapeutics, and progression of esophagogastric polyps founded in children addressed to our pediatric gastroenterology unit were studied.

The authors encountered 3 lesions in teenagers aged 13 to 17 years two males (2M), from a total number of 2140 upper digestive endoscopies (0.14%). All patients presented with pirosis, epigastric pain, and vomits; one of the children had end-stage renal disease and Kabuki syndrome. Endoscopic and pathologic findings were 2 esophageal polyps, an inflammatory one, and another containing goblet cells and a double-headed hyperplastic gastric polyp. Two patients received proton pump inhibitors without any improvement in subsequent endoscopic evaluations.

The difficulties related to age group, underlying conditions, debatable response to acid suppression, and limited experience in pediatric therapeutic endoscopy selected significantly the effectiveness of treatment.

The rarity of these lesions requires an individualized management, the endoscopic diagnostic, and therapeutic gesture depending on the symptoms, type, location, comorbidities, and team experience.

## INTRODUCTION

The polyps of the upper digestive tract have not been yet well described in the pediatric population; literature reports only isolated clinical cases or small series of patients.^1–3^ In children, the clinical features of gastrointestinal polyps depends on location and number of lesions. Considering the rarity of these lesions, the management of this patients is an increasing challenge for pediatric gastroenterologists and usually involves a multidisciplinary approach, especially in children with polyposis syndromes or a family history of gastrointestinal polyps.^4^

## BACKGROUND

Esophageal and gastric polyps are rare lesions in pediatric practice, being encountered in less than 1% of upper gastrointestinal endoscopies performed in children.^1–3,5^ These pseudotumoral formations can be identified using an imagistic (barium meal and computed tomography) or an endoscopic approach. Esophageal polyps are frequently associated with gastroesophageal reflux disease, hiatal hernia, Barrett esophagus, eosinophilic esophagitis, Crohn disease, or type 1 neurofibromatosis, whereas gastric polyps are associated with *Helicobacter pylori* infection and chronic proton pump inhibitors (PPIs) use.^4–8^ Most lesions are asymptomatic, founded incidentally during endoscopy; however, depending on their size and location, they can sometimes cause chest pain, dysphagia, early satiety, nausea, vomiting, epigastric pain, gastrointestinal bleeding, or even obstructive phenomena.^3,5^ The management of gastrointestinal polyps in children and teenagers is not thoroughly standardized; determining the underlying condition is the crucial step to take.^9–11^ Depending on the technical facilities and the skills of the surgical team, such polyps can be endoscopically or surgically removed. The aim of this article is to characterize the incidence, clinical features, histologic aspects, and therapeutic options in children with esophageal polyps.

## MATERIALS AND METHODS

We reviewed the clinical records, endoscopic charts, and pathology reports from all children that underwent upper digestive endoscopies in our pediatric gastroenterology unit in a 5 years period, from July 2010 to June 2015. All endoscopies were performed by the same team and all specimens and slides were examined by the same experienced pathologist.

## CASE SERIES

We found 3 cases in adolescents aged 13 to 17 years old, two males and a female from a total number of 2140 upper digestive endoscopies performed in our service in the mentioned period (0.14%).

### Clinical Report 1

**FIGURE 1:**
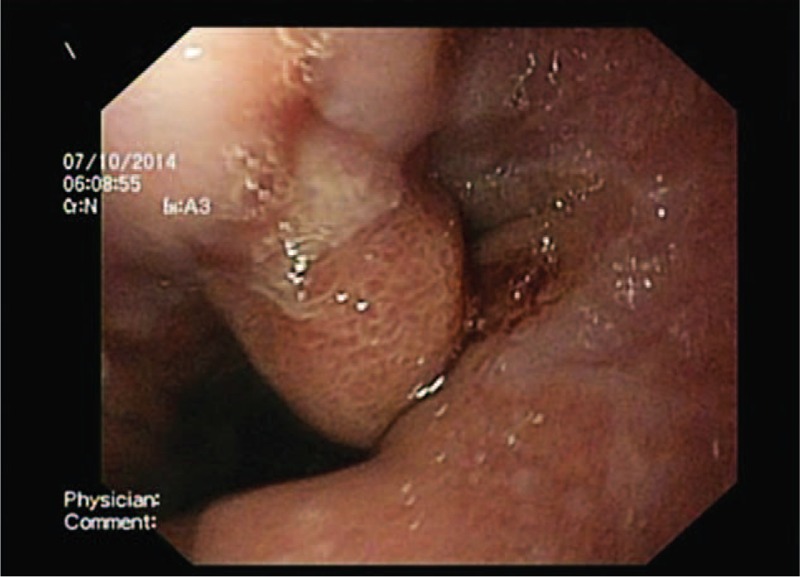
Case number 1: Esophageal inflammatory sessile polyp in a 17-year-old boy (endoscopic image).

A 17-year-old boy, with a 3-year history of pyrosis and epigastric pain, occasionally treated with PPIs and antispasmodics, was referred to our service. Physical findings were normal except epigastric pain. Laboratory data were within normal ranges. After the informed consent was obtained from his parents, he underwent an upper digestive endoscopy, which found mild erosive esophagitis (grade A in Los Angeles classification) and a sessile polyp with a diameter of 1 cm, next to the gastroesophageal junction, antral gastritis, and mucosal erythema in the first part of the duodenum (Figure 1). No association with *H. pylori* was found.

### Clinical Report 2

**FIGURE 2:**
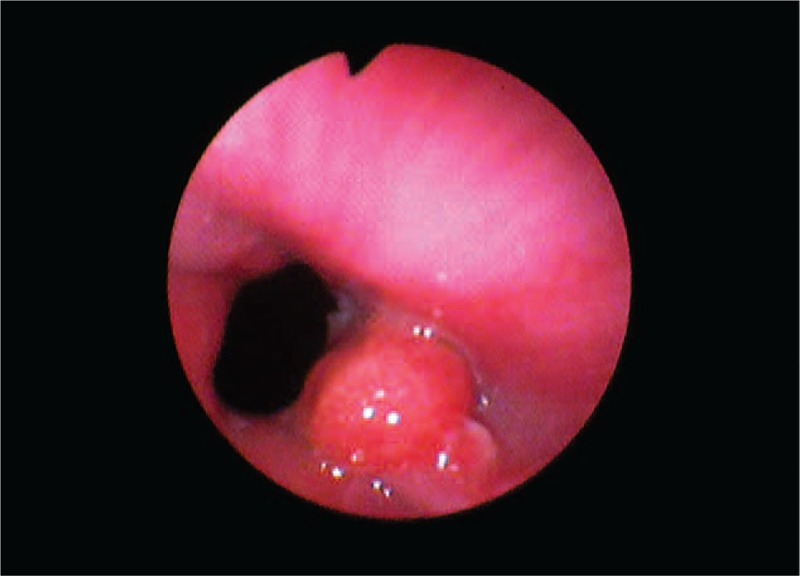
Case number 2: Esophageal sessile polyp in a 13-year-old girl (endoscopic image).

A 13 year-old girl previously hospitalized with unspecified gastroesophageal features in a county hospital without endoscopy service accused an exacerbation of epigastric pain in the last 3 months despite PPIs administration and was directed to our unit. Clinical findings included mild epigastric pain and eructations. Laboratory data were unmodified. Informed consent was given by her parents. Esophagogastroduodenoscopy showed a 7-mm diameter esophageal sessile polyp situated next to the cardia (Figure 2). Other endoscopic findings included mild esophagitis (grade A in Los Angeles classification) and antral gastritis. *Helicobacter pylori* testing was negative.

### Clinical Report 3

**FIGURE 3:**
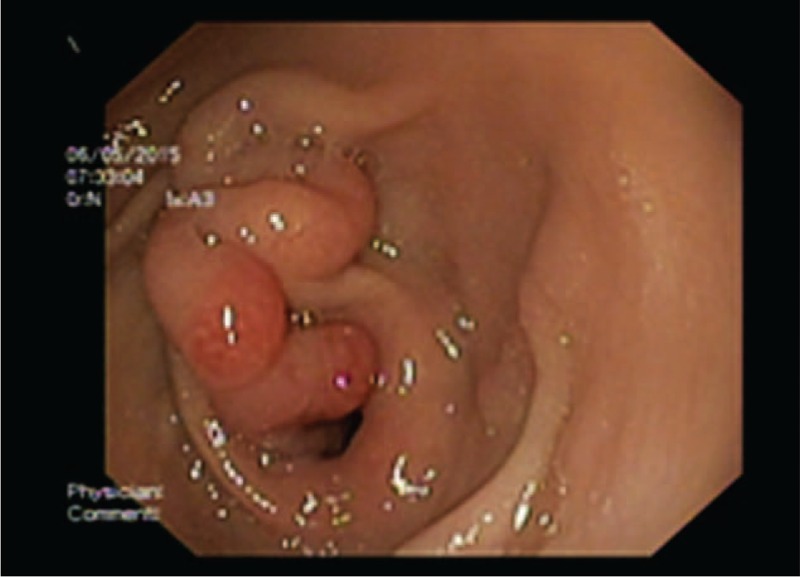
Case number 3: Giant antral fold with a double-headed polyp (endoscopic image).

A 15-year-old boy was admitted for epigastric pains and incoercible vomits. Medical history revealed complex pathology as Kabuki syndrome and end-stage renal disease (the patient being into a peritoneal dialysis program). Laboratory data showed very low glomerular filtration rate, moderate chronic anemia, severe azotemia, metabolic acidosis, hyperkalemia, hyperphosphatemia, and hypocalcemia. An informed consent was obtained from his parents. Endoscopy found a normally appearance of antral mucosa and a giant mucosal fold located next to the pylorus with a double-headed polyp (the heads were approximately 1–1.5 cm in size), one head on the gastric part of the fold and the other protruding intermittently into the duodenum (Figure 3).

**FIGURE 4:**
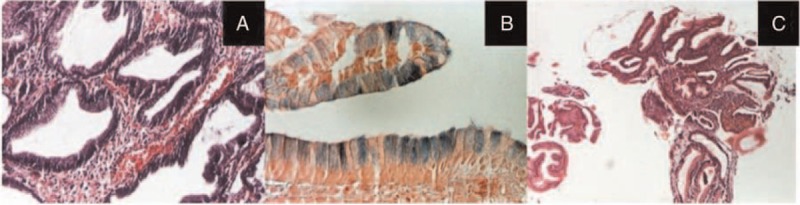
Case number 2 (A and B): Proliferation of the surface and glandular epithelium placed on conjunctival and vascular axis with a mucous secretion, moderate inflammatory infiltrate, and the presence of intracytoplasmic vacuoles positive for Alcian blue stain highlighting acid mucopolysaccharides (A: Hematoxylin-Eosin (HE) ×100, B: Alcian blue stain ×200); case number 3 (C): gastric antral mucosa with proliferation of surface and glandular epithelium and inflammatory infiltrates in corium with rare neutrophils and eosinophils (HE ×40).

In the first case, histopathology described an inflammatory polyp characterized by hyperplastic epithelium with diffuse inflammation in stroma on a reflux esophagitis pattern, without findings of eosinophilic esophagitis. In the second case, pathology revealed a proliferation of the surface and glandular epithelium placed on conjunctival and vascular axis, moderate inflammatory infiltrate, and the presence of columnar epithelium of intestinal type with goblet cells (distended, mucin-filled cytoplasm with a barrel-shaped configuration) positive for Alcian blue stain highlighting acid mucopolysaccharides (Figure 4A and B). The gastric polyps founded in the third patient were hyperplastic lesions associated with *H. pylori* infection (Figure 4C).

The first 2 patients were dismissed with PPI treatment recommendation; after 2 months, the follow-up endoscopy showed no significant difference in the macroscopic appearance of the lesions. In the third patient, triple antibacterial therapy was started in adapted doses considering the end-stage renal disease. The first patient turned 18 years (age limit for admission in pediatric services in our country) so he was directed to an adult gastroenterology unit for endoscopic surveillance and further decision on endoscopic removal of the polyp. In the second case, endoscopic surveillance was decided and continues actually twice a year. In the future, endoscopic polypectomy in collaboration with an adult endoscopist might be considered. In the third case, we could not perform any therapeutic gesture because of multiple comorbidities of the patient and his terminal status.

## DISCUSSION

Esophageal polyps are inflammatory (usually located in the middle or distal third of the esophagus) and fibrovascular (originating from the cervical esophagus). Authors described also the so-called inflammatory polyp-fold complex, squamous papillomas, and hamartomatous lesions. Gastric polyps are hyperplastic inflammatory, hamartomatous, (Peutz–Jeghers polyp, juvenile polyp, Cronkhite–Canada syndrome, Cowden disease), adenomatous (may occur in familial adenomatous polyposis), fundic gland polyps, and heterotopic lesions.^4,8,12^ In our series, we found 3 lesions, corresponding to an estimated frequency of 0.14%, which is consistent with some reports.^1–3^ In all patients, the main indication for upper digestive endoscopy was vomiting associated with other complaints, such as pyrosis and epigastric pain. The first one was an inflammatory lesion that we ascribed to a gastroesophageal reflux disease according to patient's clinical history and endoscopic findings (grade A esophagitis). As other authors had reported, we found that acid suppression therapy led only to a histologic improvement of the esophagitis without a significant change in size or appearance of the esophageal polyp. In the second case, the lesion consisted in a proliferation of the surface and glandular epithelium of intestinal goblet cells; because in other biopsy specimens we did not find any other histologic aspect suggestive for Barrett esophagus and the patient had only a short clinical history, we presumed that goblet cells are embryonic remnants of ciliated columnar epithelium described in children.^13^ In the third patient, the histology of both heads revealed the same aspect of hyperplastic lesions associated with *H. pylori* infection. In adult studies, hyperplastic polyps are more frequent compared with fundic gland polyps in regions where *H. pylori* infection is common. In contrast, in Western countries, where the prevalence of the bacteria is lower and there is a high consumption of proton pump inhibitors, the most commonly encountered polyps are fundic gland ones.^14–16^ As other authors stated, the first-line treatment in inflammatory esophageal polyps is antisecretory therapy based on PPIs.^1,2,4,16^ In our series of cases, the patients were unresponsive. The first two children were reassessed endoscopically and histologically after 2 months and had the same endoscopic appearance of the polyps, although in this interval, the antisecretory treatment was continuously followed in (the) appropriate dosage.

In this context, our opinion is that that endoscopic or surgical polypectomy must be considered in the future. Decision of endoscopic removal will be taken in multidisciplinary team including an adult gastroenterologist. Team work is also essential because of the modest development of therapeutic pediatric endoscopy in our region. To our knowledge, this is the first report on upper digestive polyps in children from our country. Nevertheless, this study has several limitations, the main one being that is a single-center study; future research in collaboration with other pediatric gastroenterology and endoscopy services is required.

## CONCLUSIONS

The rarity of these pathologic conditions requires an individualized therapeutic approach. The opportunities for therapeutic endoscopic intervention depend on the type, the location, the size of the polyp, basic condition, and comorbidities. Clinical, endoscopical, and histologic monitoring in all cases as well as “continue monitoring” in adult services after the age of 18 are required in all children with isolated polyps of the upper digestive tract.
